# Characterization of the cecum microbiome from wild and captive rock ptarmigans indigenous to Arctic Norway

**DOI:** 10.1371/journal.pone.0213503

**Published:** 2019-03-11

**Authors:** Alejandro Salgado-Flores, Alexander T. Tveit, Andre-Denis Wright, Phil B. Pope, Monica A. Sundset

**Affiliations:** 1 Department of Arctic and Marine Biology, UiT The Arctic University of Norway, Langnes, Tromsø, Norway; 2 College of Agricultural, Human, and Natural Resource Sciences, Washington State University, Pullman, Washington, United States of America; 3 Faculty of Chemistry, Biotechnology and Food Science, Norwegian University of Life Sciences, Aas, Norway; The University of Sydney, AUSTRALIA

## Abstract

Rock ptarmigans (*Lagopus muta*) are gallinaceous birds inhabiting arctic and sub-arctic environments. Their diet varies by season, including plants or plant parts of high nutritional value, but also toxic plant secondary metabolites (PSMs). Little is known about the microbes driving organic matter decomposition in the cecum of ptarmigans, especially the last steps leading to methanogenesis. The cecum microbiome in wild rock ptarmigans from Arctic Norway was characterized to unveil their functional potential for PSM detoxification, methanogenesis and polysaccharides degradation. Cecal samples were collected from wild ptarmigans from Svalbard (*L*. *m*. *hyperborea*) and northern Norway (*L*. *m*. *muta*) during autumn/winter (Sept-Dec). Samples from captive Svalbard ptarmigans fed commercial pelleted feed were included to investigate the effect of diet on microbial composition and function. Abundances of methanogens and bacteria were determined by qRT-PCR, while microbial community composition and functional potential were studied using 16S rRNA gene sequencing and shotgun metagenomics. Abundances of bacteria and methanogenic Archaea were higher in wild ptarmigans compared to captive birds. The ceca of wild ptarmigans housed bacterial groups involved in PSM-degradation, and genes mediating the conversion of phenol compounds to pyruvate. *Methanomassiliicoccaceae* was the major archaeal family in wild ptarmigans, carrying the genes for methanogenesis from methanol. It might be related to increased methanol production from pectin degradation in wild birds due to a diet consisting of primarily fresh pectin-rich plants. Both wild and captive ptarmigans possessed a broad suite of genes for the depolymerization of hemicellulose and non-cellulosic polysaccharides (e.g. starch). In conclusion, there were no physiological and phenotypical dissimilarities in the microbiota found in the cecum of wild ptarmigans on mainland Norway and Svalbard. While substantial differences in the functional potential for PSM degradation and methanogenesis in wild and captive birds seem to be a direct consequence of their dissimilar diets.

## Introduction

Ptarmigans (*Lagopus muta*) are gallinaceous birds within the subfamily *Tetraoninae*. With up to 30 different subspecies recognized, these birds show a circumpolar distribution in the northern hemisphere [1, 2]. Some physiological and phenotypical differences have been described between Svalbard (*L*. *m*. *hyperborea*) ptarmigan and rock ptarmigan from Scandinavia (*L*. *m*. *muta*), with Svalbard ptarmigans having a higher average body weight and size. In addition, Svalbard ptarmigans display striking variations in body weight and food intake throughout the year [3–5].

Despite being geographically isolated from one another, ptarmigans from Svalbard and ptarmigans in northern Scandinavia feed on diets with a similar polysaccharide composition, including a large fraction of plants rich in toxic plant secondary metabolites (PSMs) especially in periods from late fall to early summer [6–8]. Digestion can be compromised by the ingestion of PSMs, and in some cases being even toxic to the host [9]. Like other herbivores, ptarmigans have high concentrations of microbes in their main anaerobic chamber, a paired ceca protruding out from the ileo-colonic junction [10]. This cecal microbial consortium is important for the degradation of ingested plant material, yielding metabolites like Short-chain fatty acids (SCFAs) that can be absorbed by the host and used as a source of energy [10,11]. Herbivores such as the Greater Sage-Grouse (*Centrocercus urophasianus*) and woodrats (*Neotoma lepida*)) also consume diets with high levels of PSMs and possess a high proportion of PSM-degrading microbial taxa that are enriched in genes associated with the metabolism of aromatics (a key component of several PSMs) [12–14]. We therefore hypothesize that microbial groups specialized in PSM-degradation will be found in the cecum of both subspecies of ptarmigans.

In some cases a relatively high proportion of the microbiota is shared by wild and captive animals, as observed in woodrats [12]. Furthermore, studies in captive birds indicated the presence of more homogeneous cecum microbiota, presumably due to limited dietary fiber and reduced variety of PSMs compared to their free-ranging counterparts [10, 15]. We hypothesized that captive rock ptarmigans would carry different microbiota than the two subspecies of free-ranging ptarmigans due to a strong influence of diet.

The SCFAs produced during microbial polysaccharide degradation can act as substrates for methane production by methanogenic Archaea, which catalyze the last step in the anaerobic fermentation of organic polymers [16]. Methane production therefore represents a loss of metabolic energy for the host, in ruminants accounting for 2–12% of the gross energy intake (GEI) [17,18]. Avian-related methanogenesis has been neglected compared to other herbivores, with only a few studies assessing the methane production or characterizing the communities of methanogenic Archaea [19–23]. More research on the role of methanogenic Archaea and methane production would allow a better evaluation of the digestive physiology in birds.

A detailed description of the cecal microbiota (bacteria and archaea) from two subspecies of ptarmigans feeding on late autumn / winter natural plants on Svalbard and in northern Norway is presented. Moreover, the gut microbiota of wild and captive ptarmigans was investigated to identify how diet composition influences the microbial diversity and associated metabolic processes like PSM-degradation, hydrolysis of polysaccharides, fermentation, and methanogenesis.

## Methods

### Ethics statement

Rock ptarmigans (*Lagopus muta*) in Norway and at Svalbard are not an endangered or a protected species. Samples for characterization of the cecum microbiome in wild rock ptarmigans from Svalbard (*L*. *m*. *hyperborea*) and northern Norway (*L*. *m*. *muta*) were collected as part of the legal hunt during fall/winter. Samples were imported from Svalbard to mainland Norway by permission from the Norwegian Food Safety Authority.

For photoperiodic experiments the birds were placed in indoor cages (L×D×H = 100×70×45 cm) in temperature and light controlled rooms specifically designed for chronobiology studies. The birds were visually, but not acoustically separated. Indoors, birds were kept under constant temperature (~5°C, well within the thermo neutral zone of ptarmigans) and defined light-dark (LD) regimes (e.g., short days LD 8:16; long days LD 16:8) for up to two months. Samples from captive Svalbard ptarmigans fed a diet of commercial pelleted feed, were collected after sacrificing the birds in our laboratory facility appropriate for that purpose, following the method for euthanasia by sedation with Ketamine (10–20 mg/kg) and xylazin (2–4 mg/kg) followed by cervical dislocation.

Housing and manipulation of captive ptarmigans as well as the method for euthanasia were approved by the National Animal Research Authority (FOTS_ID: 7971).

### Sampling

Free-ranging rock ptarmigans were hunted in Svalbard (SPW = **S**valbard **P**tarmigan in the **W**ild; n = 4) (78°N 16°E) and northern Norway (NPW = **N**orwegian **P**tarmigan in the **W**ild; n = 4) (Troms county, 69.81°N 18.78°E) during the official hunting season (Sep—Dec). Immediately after shooting the bird cecal contents were fixed by mixing it with 70% ethanol and kept at 4°C until DNA extraction and molecular analyses. The diet composition of Svalbard ptarmigans were determined based on crop contents as previously shown [8]. The willow species *Salix polaris* constituted the majority of the crop content in late fall, together with smaller quantities of *Saxífraga cespitosa*, *Poa alpina* leaves, and Drada *spp*. [7]. No detailed description of the crop contents for the rock ptarmigans from mainland Norway used in this study was available. However, a description of the natural diet of rock ptarmigans during late fall/winter in northern Finland has been reported previously [6].

Two groups of captive Svalbard rock ptarmigans were artificially exposed to two different photoperiods mimicking light conditions during summer seasons (long photoperiod = 24 hours of light) (CP24h = **C**aptive **P**tarmigan **24** hou**rs; n = 4**) or winter seasons (short photoperiod = 6 hours of light) (CP6h = **C**aptive **P**tarmigan **6** hou**rs; n = 4**). The treatment lasted two months and was included to assess the effect of diet independent seasonal variations on the cecal microbiome, as previously observed in other animals [24]. Captive birds were fed a commercial pelleted feed (Rypefor / Ptarmigan feed) (Norgesfor AS, Oslo, Norway) with the following composition: crude protein (10.4%), crude fat (6.2%), crude ash (8.3%), crude fiber (14.4%), and supplemented mineral and essential amino acids. Cecal contents were collected immediately after slaughter and kept frozen at -80°C, until DNA extraction and molecular analyses.

### DNA extraction

The genetic material (DNA) was extracted (for the amplicon and metagenomics sequencing studies) for each individual sample following the Repeated Beat Beating plus Column (RBB+C) method [25], and the resulting DNA was quantified using a NanoDrop 2000c spectrophotometer (Thermo Scientific, US).

### Quantitative real-time PCR

Estimations of the cell density for methanogens and bacteria found in the different samples were carried out by quantitative real-time PCR (qRT-PCR). External standards were created for both microbial groups. Briefly, standards for methanogens were produced using purchased purified DNA previously extracted from a pure culture of *Methanobrevibacter ruminantium* (ATCC 35063) (DSMZ, Leipzig, Germany). The concentration of DNA was measured with NanoDrop and used to estimate the cell concentration for the axenic culture (3.97 × 10^7^). Log serial dilutions of the DNA extract were subsequently prepared from an estimated initial cell concentration of 3.97 × 10^7^ to 3.97 × 10^3^ cells per mL of *Mbr*. *ruminantium*. Bacterial external standards were performed as described by Denman and McSweeney [26], using DNA from a pure culture of *Ruminococcus flavefaciens* (Sijpesteijn 1948) (DSMZ, Leipzig, Germany). Bacterial cells were anaerobically grown using a commercial culture medium (DSMZ medium 436) (DSMZ, Leipzig, Germany), counted microscopically and serially diluted from a range of 2.26 × 10^8^ to 2.26 × 10^4^ cells per mL. The primers for qRT-PCR were previously described in [27]. Reactions were performed in a CFX384 Touch Real-Time PCR detection system (BioRad). A PCR mix consisting of 12.5 μL of SsoAdvanced Universal SYBR Green Supermix (BioRad, Hercules, CA, USA), 1.25 μL of each primer (400 nM), 5 μL DNA template (2 ng/μL), and distilled water to a 25 μL final volume was used. Experimental conditions were adjusted based on the DNA target (S1 Table), followed by a melting curve analysis to test for primer specificity and DNA contamination. Threshold cycles (C_t_) values were automatically calculated by BioRad CFX software (v3.0), and PCR efficiency was computed based on the logarithmic fraction of the resulting sigmoid-shaped curve [28]. Triplicates were run for each reaction and only those yielding the highest efficiency (R^2^≥0.996) were included in the analysis.

### 16S rRNA gene PCR amplification

The 16SrRNA gene for archaea and bacteria was amplified in an Eppendorf Mastercycler Gradient (Eppendorf AG, Hamburg, Germany) with a 25 μL reaction volume containing 12.5 μl of iProof High-Fidelity Master Mix (BioRad), 1 μl of each primer (400 nM), 1 μl of DNA template, and 1.25 μl of dimethyl sulfoxide (DMSO) to increase reaction efficiency. Amplification of 16S rRNA genes from archaea and bacteria was performed with the archaeal primer set 340F and 1000R [29], giving a 650 bp amplicon product, and the bacterial primer sets 27F and 515R [30,31], yielding a 500 bp amplicon product. Both primers sets contained the following complements in addition to their primer sequence: the Life Sciences primer A and B sequences necessary for pyrosequencing, and an 8-nt Multiplex Identifier (MID) included only in the reverse primer [32], which is used for downstream sample identification. Experimental conditions for both archaea and bacteria were previously described in [27]. The resulting PCR products were checked on a 1.5% agarose gel and the DNA concentration was measured with a Qubit fluorimeter (Invitrogen). Due to the lack of amplification with primers targeting the archaeal 16S rRNA gene, some samples within the NPW group were discarded from the analyses (NPW2 and NPW5). Samples were pooled in equimolar amounts, run in an agarose gel, excised and purified using NucleoSpin Gel and PCR Clean-up kit (Macherey-Nagel). The resulting purified, pooled DNA was stored at -20°C until sequencing. Sequencing was performed with a 454/Roche GS FLX instrument, using LIB-L chemistry, at the Norwegian Sequencing Centre (NSC), in Oslo.

For captive ptarmigans, characterization of the cecal microbiota was performed using prokaryotic (16S) and eukaryotic (18S) small subunit (SSU) rRNA genes retrieved from the metagenomics dataset (see ‘Sequence analysis’ below).

### Metagenomics analysis

For metagenomics analyses samples from wild (SPW and NPW) and captive (CP6hr and CP24hr) ptarmigans were used, giving a total of 16 samples (four per group). DNA extraction was performed as described above. Non-amplified DNA extracts were checked in a 1% agarose gel, and stored at -20°C prior to sequencing.

The sequencing service was provided by the Norwegian Sequencing Centre (www.sequencing.uio.no), a national technology platform hosted by the University of Oslo and Oslo University Hospital and supported by the "Functional Genomics" and "Infrastructure" programs of the Research Council of Norway and the Southeastern Regional Health Authorities. Libraries were prepared using TruSeq PCR-free reagents (Illumina, San Diego, CA) according to manufacturer’s instructions. Sequencing was performed on an Illumina HiSeq 3000 with 150 bp paired end reads according to manufacturer's instructions. Image analysis and base calling were performed using Illumina's RTA software version 2.7.6 and bcl2fastq v2.17.1.14. Reads were filtered to remove those with low base call quality using Illumina's default chastity criteria.

### Sequence analysis

Bacterial and archaeal 16S rRNA reads from samples of wild ptarmigans were processed using the Quantitative Insights into Microbial Ecology (QIIME) software (v1.9.1) [33]. Sequences with an average quality score above 25 were selected and trimmed to 650 bp and 500 bp length for archaea and bacteria, respectively. Reads were clustered into Operational Taxonomic Units (OTU) based on a 97% similarity criterion, discarding any putative chimeras, using the QIIME-incorporated version of USEARCH (v5.2.236). The most abundant sequence in each OTU was used as representative in downstream analyses. Representative sequences from each OTU were aligned against the Greengenes reference database (release date October 2010; http://greengenes.lbl.gov/) and taxonomically annotated as described in Salgado-Flores et al. [27]. Weighted UniFrac distance matrices were calculated for pairwise comparisons (beta-diversity) of subsampled dataset adjusted to the one with the lowest counts to avoid any potential bias [34] (S2 Table). Weighted UniFrac distances were finally used to create Principal Coordinate Analysis (PCoA) plots. A network map comparing the amount of shared OTUs between pairs of samples were computed with the make_otu_network.py script in QIIME, and visualized with Cytoscape (v3.1.1) [35].

Shotgun metagenomic sequencing data was analyzed as previously described [36] (S3 Table). Briefly, paired-end sequences were assembled with Pandaseq (v2.9) [37], with a 10 bp minimum overlap. Assembled sequences were preprocessed with Prinseq lite (v0.20.4) [38]; sequences with an average quality value below 20 and more than five unambiguous bases were removed, and all but one sequence of identical sequence pools were discarded to avoid any bias due to artificial replication during sequencing. A 5,000,000 sequences subset from each sample was used for downstream analysis. DNA sequences were used as query for BLASTX analysis using DIAMOND alignment tool (‘—more_sensitive’ option; e-value 1e-4) against the RefSeq-protein database from NCBI [39]. The same subset was screened using the ‘hmmsearch’ tool within the package HMMER [40] with hidden markov models (HMMs) for protein families (Pfam release 30, http://pfam.janelia.org]. All searches were carried out on the High Performance Computing (HPC) Stallo at UiT-The Arctic University of Norway (http://docs.notur.no/uit). Output files from Diamond were uploaded and visualized in MEGAN Community Edition (default parameters: minimum bit score of 50, minimum node support of 1) [41].

For the taxonomically comparisons between captive and wild ptarmigans, 16S and 18S rRNA gene sequences were extracted using the ‘SortMeRNA’ tool and Silva rRNA gene database 123 (July 23^rd^, 2015) [42]. The extracted SSU rRNA gene reads were taxonomic annotated by comparison to the SilvaMod rRNA gene reference database using the online version of the LCA Classifier available at the Classification Resources for Environmental Sequence Tags (CREST) website (minscore: 150) (http://apps.cbu.uib.no/crest/lcaclassify) [43].

### Statistics

For qRT-PCR analysis, two-tailed Student’s t-test was applied for pair-wise comparisons between wild and captive groups’ data for methanogens and bacteria. Moreover, one-way analysis of variance (ANOVA) tests were performed for multi-group analysis for bacteria and methanogens qRT-PCR data. When statistical significance was observed, Tukey´s range test was performed to obtain further verification at a group level [44]. All the aforementioned tests were performed with ‘R’ statistical package [45]. A non-parametic PERMANOVA test (999 permutations), from the package ‘vegan’ in the ‘R’ tool included in QIIME, based on weighted UniFrac distances, was run to test significance for any difference between Svalbard and northern Norway ptarmigans cecum dataset (bacteria and archaea). Bar plots representing relative abundances (normalized to the total reads for each group) for the different KEGG categories were compared with the STAMP software [46, 47]. Statistical comparisons between functional categories were performed using White’s non-parametric *t*-test [48], corrected with Benjamini–Hochberg False Discovery Rate correction. Only those categories yielding significant differences were represented.

## Results

### Quantitativereal time PCR analysis

**Fig 1:**
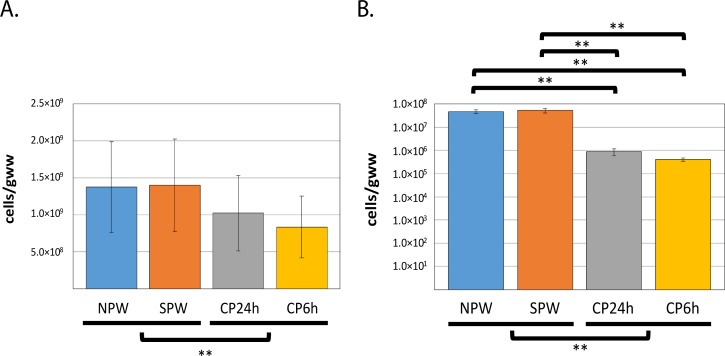
Concentration of bacteria and methanogens in the cecum of wild and captive ptarmigans. Bacteria (A) and methanogens (B) populations were estimated by qRT-PCR. Total counts are presented as number of cells per gram of wet weight (cells/gww). Bacterial counts were plotted using a linear scale whereas for methanogens a logarithmic scale was applied for better visualization. Wild rock ptarmigan northern Norway (NPW) (n = 4); wild Svalbard rock ptarmigan (SPW) (n = 4); captive Svalbard ptarmigan exposed to long (CP24h) (n = 4) or short (CP6h) (n = 4) photoperiods. Pair-wise statistical comparisons were calculated with a two-tailed Student’s *t*-test. Statistical analysis for multiple groups were calculated with ANOVA tests. Tukey´s range test was applied for further verification at group level * (*p*-value<0.05); ** (*p*-value<0.01).

We estimated the cell densities of Bacteria and methanogenic Archaea in order to assess the importance of the methane producing microbial community in comparison to the total bacterial community. Overall, bacterial mean counts were approximately 20 times higher than for methanogenic Archaea (1.40×10^9^ vs 4.73×10^7^–5.31×10^7^16S rRNA gene copies/gww) (Fig 1). No significant differences were observed between the mean cell counts of bacteria (Student’s *t*-test: p = 0.927) or methanogens (p = 0.716) in SWP and NWP. Captive birds possessed significantly lower mean cell densities for both bacteria (CP24h: 1.02×10^9^; CP6h: 8.35×10^8^ 16S rRNA gene copies/gww; ANOVA: p-value = 0.01) and methanogens (CP24hrs: 8.89×10^5^; CP6hrs:4.05×10^5^ 16S rRNA gene copies/gww; ANOVA: p-value<0.01) compared to wild ptarmigans (Fig 1). However, when comparisons were performed based on individual groups (e.g. SPW versus CP24h) and not by living conditions (i.e. captive versus wild), no significant differences in bacterial mean cell densities were observed between wild and captive ptarmigans (Fig 1; ANOVA: p-value = 0.09). Methanogens mean cell densities were significantly different between all the sub-groups for captive and wild ptarmigans (Fig 1; ANOVA: p-value<0.05; Tukey´s: p<0.05). Furthermore, no significant differences in bacterial or methanogen abundances were observed between captive ptarmigans exposed to different light regimes (methanogens: p = 0.189; bacteria: p = 0.412) (Fig 1).

### Taxonomic identification

#### Bacterial 16 rRNA gene

**Fig 2:**
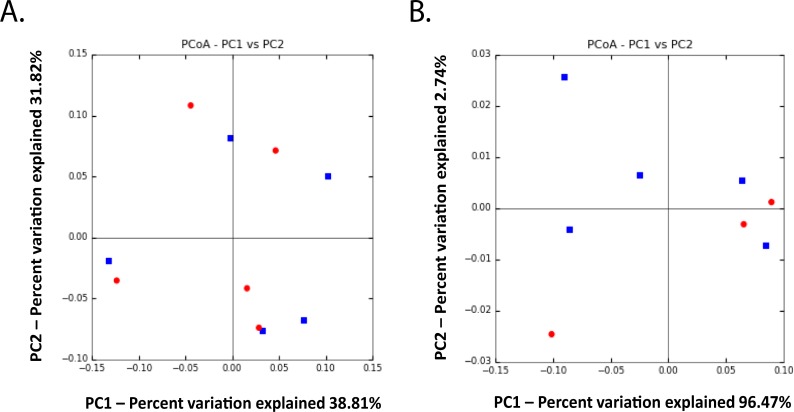
Principal coordinate analysis (PCoA) of microbial communities from the cecum of wild ptarmigans based on 16S rRNA amplicon sequencing data. Plots were generated based on weighted UniFrac distance matrices. A) PCoA plot of bacterial community structure; B) PCoA plot of archaeal community structure. Blue square: Svalbard ptarmigan. Red circle: rock ptarmigans northern Norway.

A total of 100,817 and 88,259 bacterial 16S rRNA gene sequences (average length 500 bp) were obtained from the Svalbard ptarmigans and Norwegian wild ptarmigans, respectively. Clustering of OTUs resulted in a total of 1,939 chimera-free OTUs at 97% sequence similarity, from which 1,754 (90.4% total OTUs) were shared between the groups of wild ptarmigans. The bacterial communities of SWP and NWP did not separate in weighted UniFrac-based PCoA plots (Fig 2A). Further testing confirmed that there is no significant difference in the bacterial communities between wild ptarmigans from Svalbard and mainland Norway (pseudo-F = 0.823; p-value = 0.79).

The same dominant phylotypes were observed in SWP and NWP samples, with *Actinobacteria* (SPW: 28.1%; NPW: 27.7% of total sequences, on average), *Firmicutes* (SWP: 24%; NWP: 25.1%), *Bacteroidetes* (SWP: 18.3%; NWP: 19%), and *Synergistetes* (SWP: 10.7%; NWP: 11.1%) as the major phyla (Table 1). No significant differences were found in the relative abundances of any of these phyla, between SWP and NWP (p>0.05) (Table 1). Although largely similar, some differences between shotgun metagenomics SSU and 16S rRNA gene amplicon sequencing results were observed. Firmicutes was the dominant phylotype (SWP: 33.9%; NWP: 32.6%) instead of *Actinobacteria* (SWP: 19.3%; NWP: 19.3%).

**Table 1:** Main microbial groups identified in cecal samples from wild ptarmigans based on 16S rRNA amplicon sequencing.

Taxonomical level	Archaeal group	NPW	SPW	*p*-value
Family	*Methanocorpusculaceae*	27.8	35.4	0.738
Family	*Methanomassiliicoccaceae*	71.7	62.8	0.685
Family	*Methanobacteriaceae*	0.5	1.8	0.299
Level	Bacteria			
Phylum	*Deferribacteres*	0.2	0.3	0.690
Phylum	*Tenericutes*	1	1.4	0.461
Phylum	*Synergistetes*	11.1	10.7	0.908
Phylum	*Spirochaetes*	6.6	6.5	0.960
Phylum	*Firmicutes*	25.1	24	0.637
Phylum	*Actinobacteria*	27.7	28.1	0.870
Phylum	*Bacteroidetes*	18.9	18.3	0.733
Phylum	*Proteobacteria*	2.1	4	0.347

Statistical tests were performed using two-tailed student’s *t*-test.

Values are presented as percent of total bacterial or archaeal 16S amplicon reads in wild Norwegian rock ptarmigans (NPW) or wild Svalbard rock ptarmigans (SPW).

**Fig 3:**
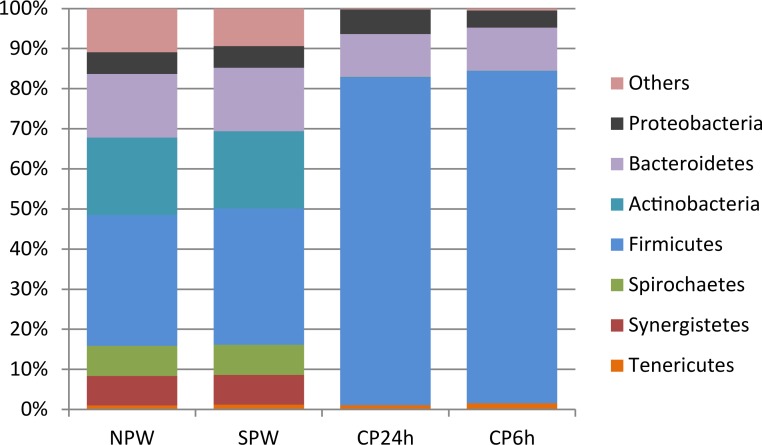
Percent relative abundance of bacterial phyla in the cecum of wild and captive ptarmigans based on shotgun metagenomic data. Bar charts illustrates the taxonomical relative diversity of bacteria at phylum level from SSU sequences benchmarked against the SilvaMod rRNA reference database as described in material and methods section. Wild rock ptarmigan northern Norway (NPW) (n = 4); wild Svalbard rock ptarmigan (SPW) (n = 4); captive Svalbard ptarmigan exposed to long (CP24h) (n = 4) or short (CP6h) (n = 4) photoperiods.

Analysis of SSU rRNA gene sequences extracted from shotgun metagenomics data showed that captive ptarmigans were dominated by few bacterial phyla: *Firmicutes* (CP24h: 81.7%; CP6h: 82.8%), *Bacteroidetes* (CP24h: 10.7%; CP6hrs: 10.7%) and *Proteobacteria* (CP24h: 6.2%; CP6h: 4.3%) (Fig 3). Similarly, only a few families dominated: *Ruminococcaceae* (CP24h: 41.6; CP6h: 42.8), *Lachnospiraceae* (CP24h: 29.6; CP6h: 31.2), and *Enterobacteriaceae* (CP24h: 5.5; CP6h: 3.7). No significant differences in bacterial groups between captive ptarmigans exposed to different photoperiods were observed (Fig 3).

#### Archaeal 16 rRNA gene

In total, 109,506 16S rRNA sequences (average length 635 bp) were obtained from cecal samples of wild Svalbard ptarmigans and Norwegian ptarmigans after quality filtering. We identified a total of 67 OTUs (97% threshold), of which 54 were shared by SWP and NWP. All OTUs were assigned to one single phylum, *Euryarchaeota* (Table 1). Similar to bacteria, beta-diversity comparisons based on weighted UniFrac distances showed that the SWP and NWP samples grouped together (Fig 2B). In order to test if there were any differences between the archaeal community profiles a PERMANOVA test with 999 permutations was performed, and no significant differences were observed (pseudo-F = 0.23; p-value = 0.76).

The newly characterized family *Methanomassiliicoccaceae* (SWP: 62.8% of total sequences, on average; NWP: 71.7%) was the dominant archaeal taxon. Genera belonging to the family *Methanocorpusculaceae* constituted the other major archaeal group (SWP: 35.4%; NWP: 27.8%). No significant differences were found in the relative abundances of these taxa between SWP and NWP (p>0.05) (Table 1). Within captive ptarmigans, results based upon shotgun metagenomics sequencing data indicated *Methanobacteriaceae* as the only archaeal family identified.

### Metagenomics analysis

#### PSMs degradation and methanogenesis pathways

**Fig 4:**
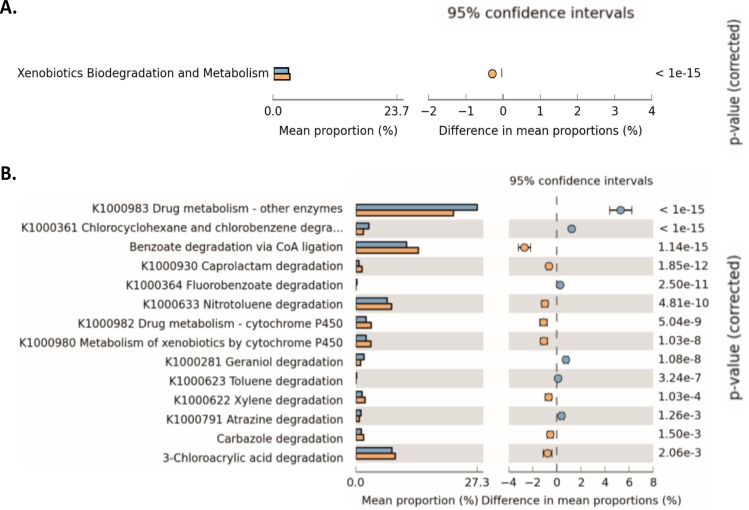
Bar plots of KEGG Orthologous functions for xenobiotics degradation in the ceca of wild and captive ptarmigans. Comparisons were carried out with calculated mean values of shotgun metanogenomic sequences from captive and wild ptarmigans. The mean proportion of total sequences is represented allocated to: A) The whole KEGG class “Xenobiotics and Biodegradation Metabolism”; B) KEGG pathways within the “Xenobiotics and Biodegradation Metabolism” class. Only those categories showing significant differences between both groups of ptarmigans are presented. Statistical tests were performed using the White’s non-parametric *t*-test applying the Benjamini-Hochberg False Discovery Rate correction. KO codes are presented in the beginning of each name tag (if any). Wild ptarmigans (yellow); captive Svalbard ptarmigan (blue).

**Fig 5:**
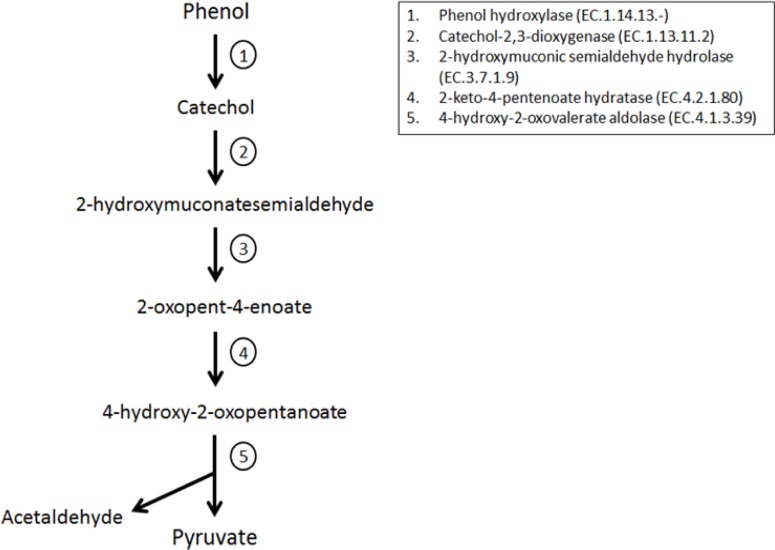
Schematic representation of a complete pathway leading to the conversion of phenol into pyruvate in wild rock ptarmigans. Full name and KEGG entries for the enzymes involved at each step (1–5) are given in the legend box. Only a few of these genes were present (or at a minor proportion) in captive ptarmigans. A similar pathway has been previously identified in the cecum of Greater sage-grouse [14]: 1. Phenol hydroxylase (EC.1.14.13.-); 2. Catechol-2,3-dioxygenase (EC.1.13.11.2); 2-hydroxymuconic semialdehyde hydrolase (EC.3.7.1.9); 2-keto-4-pentenoate hydratase (EC.4.2.1.80); 4-hydroxy-2-oxopentenoate (EC.4.1.3.39).

In order to obtain insights on the effects of diet on the genetic repertoire of the microbiome from wild and captive ptarmigans, genes encoding enzymes for PSM detoxification were mapped using Kyoto Encyclopedia of Genomes and Genes (KEGG). Xenobiotics degradation, a category comprising genes for the degradation of components found in several PSMs were more abundant in wild than captive ptarmigans (Fig 4A). Genes for pathways of chloroacrylic acid, benzoate, and nitrotoluene degradation were the most abundant sub-category of xenobiotics degradation in both wild and captive ptarmigans, and were significantly higher in wild than captive ptarmigans (Fig 4B). Furthermore, the relative abundance of genes for the degradation of aromatic compounds such as caprolactam (x2.19-fold), xylene (x1.77-fold), carbazole (x1.69-fold), were significantly more abundant in wild than captive ptarmigans (Fig 4B). Genes for the degradation of PSMs (xenobiotics KEGG category) in our wild ptarmigans were assigned to the phylum *Firmicutes* (families *Clostridiaceae*, *Lachnospiraceae*, and *Eubacteriaceae*), *Bacteroidetes* (genus *Bacteroides*), and *Actinobacteria* (genera *Eggerthella* and *Slackia*). The wild ptarmigan microbiota also carried genes specific for a metabolic pathway leading to the conversion of phenol and catechol to pyruvate (Fig 5). Genes for the full metabolic pathway were not present in the microbiota of captive ptarmigans.

Methanogenesis plays an important role in anaerobic fermentation by catalyzing its last steps by using waste products from microbial metabolism as substrates (e.g. SCFAs) [16]. However, methane production represents an energy loss for the host [17,18]. Genes involved in methane production were screened to obtain a better understanding on the microbial fermentation in the ceca of our wild ptarmigans. Methyl coenzyme M reductase [EC:2.8.4.1], a gene encoding the key terminal enzyme of methanogenesis, was present at an average relative abundance of 0.01% in both SWP and NWP (Table 2). Genes encoding enzymes involved in hydrogenotrophic and methylotrophic methanogenesis were also identified (Table 2; S1 Fig). Overall, higher relative abundances of genes for methanogenesis pathways were observed in wild ptarmigans (x1.7). Despite the detection of methanogenic Archaea in the SSU rRNA datasets, no genes for the methyl coenzyme M reductase were found in the captive ptarmigans (Table 2).

**Table 2:** List of KEGG genes involved in ‘methane metabolism’. Data is represented as the proportion of sequences assigned to each KEGG pathway in relation to total hits.

KEGG pathway	Captive	Wild	*p*-value
K1000680 Methane metabolism	0.053	0.090	<0.001
K00018 glycerate dehydrogenase [EC:1.1.1.29]	0.041	0.081	<0.001
K00058 D-3-phosphoglycerate dehydrogenase [EC:1.1.1.95]	0.004	0.001	0.009
K00121 S-(hydroxymethyl)glutathione dehydrogenase / alcohol dehydrogenase [EC:1.1.1.284 1.1.1.1]	0.004	0.001	0.003
Formate dehydrogenase	0.015	0.030	0.002
Pyruvate ferredoxin oxidoreductase	0.060	0.107	<0.001
K00194 acetyl-CoA decarbonylase/synthase complex subunit delta [EC:2.1.1.245]	0.026	0.004	<0.001
K00399 methyl-coenzyme M reductase alpha subunit [EC:2.8.4.1]	0.000	0.009	<0.001
K00440 coenzyme F420 hydrogenase subunit alpha [EC:1.12.98.1]	0.002	0.004	0.026
K00600 glycine hydroxymethyltransferase [EC:2.1.2.1]	0.096	0.140	<0.001
K00625 phosphate acetyltransferase [EC:2.3.1.8]	0.066	0.086	<0.001
K00850 6-phosphofructokinase 1 [EC:2.7.1.11]	0.231	0.164	<0.001
K00863 dihydroxyacetone kinase [EC:2.7.1.29]	0.005	0.002	<0.001
K00865 glycerate kinase [EC:2.7.1.31]	0.043	0.020	<0.001
K00925 acetate kinase [EC:2.7.2.1]	0.121	0.139	0.001
K01007 pyruvate, water dikinase [EC:2.7.9.2]	0.008	0.018	<0.001
K01070 S-formylglutathione hydrolase [EC:3.1.2.12]	0.005	0.000	0.002
K01595 phosphoenolpyruvate carboxylase [EC:4.1.1.31]	0.003	0.009	0.001
K01621 phosphoketolase [EC:4.1.2.9]	0.033	0.006	<0.001
K01624 fructose-bisphosphate aldolase, class II [EC:4.1.2.13]	0.128	0.115	0.008
K01689 enolase [EC:4.2.1.11]	0.101	0.138	<0.001
K01895 acetyl-CoA synthetase [EC:6.2.1.1]	0.050	0.101	<0.001
K02203 phosphoserine / homoserine phosphotransferase [EC:3.1.3.3 2.7.1.39]	0.010	0.029	<0.001
K03388 heterodisulfide reductase subunit A [EC:1.8.98.1]	0.063	0.120	<0.001
K03518 carbon-monoxide dehydrogenase small subunit [EC:1.2.99.2]	0.024	0.019	0.023
K03781 catalase [EC:1.11.1.6]	0.015	0.000	<0.001
K03782 catalase-peroxidase [EC:1.11.1.21]	0.006	0.000	0.001
K03841 fructose-1,6-bisphosphatase I [EC:3.1.3.11]	0.009	0.004	0.023
K04480 methanol—5-hydroxybenzimidazolylcobamide Co-methyltransferase [EC:2.1.1.90]	0.000	0.003	<0.001
K05884 L-2-hydroxycarboxylate dehydrogenase (NAD+) [EC:1.1.1.337]	0.001	0.000	0.023
K05979 2-phosphosulfolactate phosphatase [EC:3.1.3.71]	0.001	0.003	0.001
K08094 6-phospho-3-hexuloisomerase [EC:5.3.1.27]	0.006	0.002	0.002
K11261 formylmethanofuran dehydrogenase subunit E [EC:1.2.99.5]	0.004	0.006	0.004
K13039 sulfopyruvate decarboxylase subunit beta [EC:4.1.1.79]	0.000	0.001	<0.001
K13831 3-hexulose-6-phosphate synthase / 6-phospho-3-hexuloisomerase [EC:4.1.2.43 5.3.1.27]	0.000	0.003	<0.001
K14080 [methyl-Co(III) methanol-specific corrinoid protein]:coenzyme M methyltransferase [EC:2.1.1.246]	0.001	0.003	<0.001
K14081 methanol corrinoid protein	0.000	0.002	<0.001
K14083 trimethylamine—corrinoid protein Co-methyltransferase [EC:2.1.1.250]	0.011	0.005	0.010

### Identification of Pfam domains involved in polysaccharide degradation

In order to detail the carbohydrate-degrading potential in wild and captive ptarmigans, the major enzymes responsible for plant polysaccharide degradation were characterized. A screening of the major glycoside hydrolases (GHs), i.e. enzymes catalyzing the hydrolysis of the glycosidic bonds in complex sugars, and other enzymes involved in carbohydrate degradation was performed (Pfam release 25, https://pfam.janelia.org). In total, genes for 89 and 91 different GHs and carbohydrate degradation families were identified in the cecal microbiome of wild and captive ptarmigans, respectively (S4 Table; S2 Fig). No differences were found in the relative abundance of the major GH families (families accounting for ≥1% of sequences assigned to a carbohydrate degrading enzymes) between wild ptarmigans from Svalbard and northern Norway (Table 3). Genes encoding enzymes associated to GH family 77, catalyzing 4-α-glucanotransferase or amylomaltase activity (http://www.cazy.org/), were the single most abundant family in wild ptarmigans (9.5% total hits). In addition, genes involved in starch metabolism (e.g. starch phosphorylase) [EC.2.4.1.1]) were also found in wild ptarmigans (Wild: 0.19% of total KEGG-annotated sequences). GH families acting on xylo-oligosaccharides with a broad repertoire of catalytic activities like β-glucosidase, α and β-galactosidase (GH1-3, GH31, GH36, GH42) and xylosidase (GH43) were abundant in wild ptarmigans and accounted, altogether, for 49.8% total hits, on average (Table 3). Genes for rhamnose (GH78) (6% of total hits) and mannose (GH92) (4.6%) degradation were also among the major GH families in wild ptarmigans (Table 3; S4 Table). Genes for GH families involved in the degradation of pectin constituents such as rhamnose and galacturonans (e.g. GH78, GH28, GH53, GH106), in wild ptarmigans shows that the microbiota has the potential for pectin degradation (Table 3; S4 Table). Moreover, genes for pectinesterase [EC.3.1.1.11], an enzyme catalyzing the hydrolysis of pectin into pectate and methanol, were present in both groups of wild ptarmigans.

**Table 3:** Major pfam families associated with polysaccharide degradation in cecal samples from wild ptarmigans.

Pfam	Family	NPW (%)	SPW (%)	Average (both)	Function	Category
PF02446.14	GH77	9.506	9.421	9.464	4-α-glucanotransferase/amylomaltase	Starch degradation
PF00933.18	GH3*	9.180	9.140	9.160	β-glucosidase	Oligosaccharide hydrolase
PF02836.14	GH2-C*	6.671	6.608	6.639	β-galactosidase	Oligosaccharide hydrolase
PF01915.19	GH3-C*	5.481	5.418	5.450	β-glucosidase	Oligosaccharide hydrolase
PF00232.15	GH1*	4.980	5.271	5.126	β-glucosidase	Oligosaccharide hydrolase
PF01055.23	GH31*	5.216	4.993	5.104	α-glucosidase	Starch degradation
PF07971.9	GH92	4.581	4.605	4.593	α-mannosidase	Oligosaccharide hydrolase
PF05592.8	GH78	4.306	4.581	4.443	α-L-rhamnosidase	Debranching enzyme
PF04616.11	GH43	4.091	4.013	4.052	arabino/xylosidases	Oligosaccharide hydrolase
PF07944.9	GH127	3.610	3.534	3.572	β-L-arabinofuranosidase	Debranching enzyme
PF02837.15	GH2-N*	3.166	3.168	3.167	β-galactosidase	Oligosaccharide hydrolase
PF00728.19	GH20	2.797	2.713	2.755	β-hexasominidase	Oligosaccharide hydrolase
PF10566.6	GH97	2.348	2.337	2.343	α-glucosidase	Oligosaccharide hydrolase
PF07470.10	GH88	2.210	2.312	2.261	d-4,5-unsaturated β-glucuronyl hydrolase	Debranching enzyme
PF07745.10	GH53	2.143	2.251	2.197	endo-1,4-β-galactanases	Endohemicellulose
PF01183.17	GH25	2.106	2.049	2.078	Lysozyme	
PF00251.17	GH32-N	2.068	2.057	2.062	Invertase	
PF02449.12	GH42*	1.953	1.977	1.965	β-galactosidase	Oligosaccharide hydrolase
PF16875.2	GH36N*	1.852	1.859	1.855	α-galactosidase	Oligosaccharide hydrolase
PF00703.18	GH2*	1.864	1.816	1.840	β-galactosidase	Oligosaccharide hydrolase
PF06964.9	Alpha-L-AF-C^a^	1.573	1.475	1.524	α-L-arabinofuranosidase	Debranching enzyme
PF03065.12	GH57	1.348	1.373	1.366	α-galactosidase/α-amylase	Oligosaccharide hydrolase
PF02922.15	CBM_48	1.277	1.331	1.304	Binding-module potentially associated to starch-debranching enzymes	Starch degradation
PF08531.7	Bac_rhamnosidase-N	1.197	1.322	1.260	α-L-rhamnosidase	Debranching enzyme
PF03629.15	SASA	1.056	1.215	1.135	Carbohydrate esterase	
PF17167.1	GH36*	1.101	1.078	1.089	α-galactosidase	Oligosaccharide hydrolase

Values are given as percent of total pfam families associated to polysaccharide degradation.

* = associated to hemicellulose degradation

GH = Glycoside hydrolase; a = alpha-L-arabinofuranoside; C = C-terminal domain; N = N-terminal domain. NPW = Wild Norwegian rock ptarmigan; SPW = Wild Svalbard rock ptarmigan.

The same genes for carbohydrate degradation dominated wild ptarmigans and captive ptarmigan metagenomes (Fig A in S2 Fig), but with differences in some categories, e.g. higher relative abundances of genes for endohemicellulose and starch degradation in wild ptarmigans compared to both captive ptarmigan groups (Fig C and D in S2 Fig).

## Discussion

### Total populations of bacteria and methanogens in cecum of wild and captive ptarmigans

The abundance of bacteria and methanogens were lower in the ptarmigans of this study than previously observed in several ruminants, and chicken [49–51] (S5 Table); however, bacterial counts were higher than described in wild seabirds [52]. Few studies have reported estimations of methanogen populations in birds, with total numbers being higher in the crop of the wild Hoatzin (*Opisthocomus hoazin*) and lower in the ceca of captive chickens than in the ceca of the ptarmigans in our study [20,21].

Significant differences were observed between wild and captive ptarmigans for both bacteria and methanogens (Fig 1). Such differences may be a consequence of commercial feed (as the one given to captive ptarmigans) being ground and pelleted, which leads to faster digestion rates and, therefore, in shorter retention times and lower microbial growth [53]. In contrast, diets possessing a higher variety of structural polysaccharides, as those presumably consumed by the wild ptarmigans, would require longer retention times for their degradation, which may lead to higher microbial growth rates for bacteria and methanogens.

Moreover, considering the important role played by anaerobic fungi in the physical disruption and degradation of fiber compounds in, for instance, the rumen [54,55], it might also be speculated that there is a higher presence of this microbial group in the cecum of wild ptarmigans. Longer feed passage rates would provide enough time to fungi to proliferate and help physically disrupt the fiber. New studies would have to integrate data from these microbes to further understand microbial fiber degradation in wild ptarmigans.

### Specific bacterial taxa may mediate PSM-detoxification in wild ptarmigans

Bacterial communities in wild and captive ptarmigans were composed differently (Fig 3). Microbiome variation between wild Japanese captive Svalbard ptarmigans have been reported previously [15]. Consistently, these wild Japanese ptarmigans and the wild ptarmigans in our study possessed similar cecal microbiota (Table 1). Genes associated with PSM-degradation in our wild ptarmigans were allocated to the bacterial phyla *Firmicutes* (families *Clostridiaceae*, *Lachnospiraceae*, and *Eubacteriaceae*), *Bacteroidetes* (genus *Bacteroides*), and *Actinobacteria* (genera *Eggerthella* and *Slackia*). Similarly, genes for xenobiotics biodegradation in the cecum of free-ranging Greater sage-grouse were assigned to the genera *Bacteroides*, *Eggerthella* and *Clostridium* [14]. These comparisons suggest that bacterial phylotypes putatively perform the same metabolic tasks in wild ptarmigans and Greater sage-grouse, PSM-degradation being driven by several bacterial groups. On the other hand, studies from the cecum and rumen microbiota in wild Japanese ptarmigans and Hawaiian goat, respectively, both animals following a diet rich in PSM, showed most of the genes involved in PSM degradation assigned to the phylum *Synergistetes* [15, 56].

Genes for the conversion of phenol and catechol into pyruvate were mostly assigned to *Firmicutes* (class *Clostridia*), *Actinobacteria* (order *Micrococcales*) and *Proteobacteria* (class *Alphaproteobacteria*) (Fig 5). Enrichment of genes encoding enzymes of this metabolic pathway was also described in the cecal microbiome of the Greater sage-grouse, mostly assigned to the genus *Anthrobacter* (family *Micrococcaceae*) [14]. Some of these genes also encode enzymes involved in xylene and benzoate metabolism, accounting for the high relative abundance of such overlapping genes in wild ptarmigans.

Chemical analysis by Hansen et al. (2011) revealed the presence of several PSMs, e.g., the two benzene ring flavonoid Catechin, in *Salix polaris* tissues [57]. High contents of PSMs in *Salix* spp. might select for bacterial groups bearing genes mediating PSMs degradation. The presence of genes encoding enzymes involved in the degradation of phenol/catechol compounds in wild ptarmigans (Fig 5), but absence in the captive group, indicates that such compounds are utilized by the wild ptarmigan cecum microbiota. Whether the intermediates in PSM degradation (e.g., pyruvate) can be used by the host remains unknown.

### Hemicellulose and starch-hydrolyzing GHs in cecum of wild ptarmigans

A broad repertoire of GHs families were found in the ceca of wild and captive ptarmigans. The total number of GHs was higher than those reported for other herbivores (e.g. bovine and reindeer rumen, hindgut fermenters, etc.) and comparable to feces from captive Asian elephants (see Table 2 in [58]) (S6 Table). The broad range of GHs, irrespective of the diet composition, for wild and captive ptarmigans suggests an inherent high richness in hydrolytic enzymes in the cecum microbiota of ptarmigans. Such diversity could allow this microbial consortium to readily adapt to a changing diet throughout seasons [8]. The presence of higher relative abundances of GHs involved in starch degradation and the lower proportions of cellulases observed in wild ptarmigans compared to the captive groups was unexpected (S2 Fig). The ingestion of a natural diet would, in theory, require a higher abundance of enzymes involved in the deconstruction of complex polysaccharides (e.g. cellulose) compared to the captive birds fed commercial pellets, whose feed is compositionally consistent as well as physically ground and more easily digestible. Interestingly, crude fiber contents in the commercial pellets used in this study (about 14% of the total chemical composition) was comparable to that reported from crop contents of wild Svalbard ptarmigans during periods when *S*. *Polaris* constituted the major food item (see Materials & Methods; [8]). The major plant species in the crop of our wild Svalbard ptarmigans was also found to be *S*. *polaris* [7]. This similarity in the crude fiber content for both dietary regimes may account for the comparable overall diversity in GHs present in wild and captive ptarmigans. Genes encoding endohemicellulases were found at a higher relative proportion in wild ptarmigans (Table 3; S2 Fig).

The GH families degrading hemicellulose compounds (GH1-3, GH8, GH10, GH16, GH26, GH31, GH36, and GH42) constituted the largest fraction of total GH genes in wild ptarmigans indicating the great potential to degrade this type of polysaccharides by the cecum microbiota (Table 3). These findings may suggest that, at least during late autumn / winter, the diet consumed by wild ptarmigans contains plants with high hemicellulose content. Our results may contradict previous reports on the diet of these birds indicating their capacity to select plants (or parts of plants) with high-nutritional value (low in fiber) in all periods [1,8]. However, the high presence of GH families encompassing genes for the degradation of starch (e.g.GH77, with amylomaltase activity [59]) (Table 3), and genes involved in starch metabolism (e.g. starch phosphorylase [EC.2.4.1.1]) also indicates that starch-containing compounds may constitute an important part of the nutrition in the wild ptarmigan diet. These findings, together with the identification of genes encoding GH families (e.g. GH78, GH28, GH53, GH106) and enzymes involved in the depolymerization of pectic compounds (e.g. pectinesterase), suggests that wild ptarmigans house a versatile microbiota that allows the digestion of plants with variable fiber contents. This versatility may be beneficial during periods when high-quality foodstuffs are scarce as in autumn / winter. Nonetheless, new studies describing the cecal metatranscriptome are necessary to find out which GH genes are expressed and the role played the different type of polysaccharides in the nutrition of wild ptarmigans.

### Archaeal taxonomy and methanogenesis in wild ptarmigans

To our knowledge, this is the first study reporting the presence of *Methanomassiliicoccaceae*-associated members in birds (Table 1). *Methanomassiliicoccaceae*-related phylotypes have been described in the gut of several hervibores (e.g. rhinoceroses, muskoxen, kangaroo, elephant, etc.) [60–62], and anaerobic digesters [63], but at a lower relative abundance than observed in wild ptarmigans. Methanol and methylamines are the main growth-substrates for members of the *Methanomassiliicocacceae* family [61, 64]. Methylamines may result from the degradation of amino acids and compatible solutes, and this methanogenesis pathway was identified in peat soil and gut samples [61, 65]. Only one gene encoding one of the key enzymes mediating methanogenesis from methylamines (trymetylamine—corrinoid protein Co-methyltransfersase [EC.2.1.1.250]) was found in captive and wild ptarmigans (captive: 0.011%; wild: 0.005% of total genes) (Table 2). However, in both cases this gene was not assigned to *Methanomassiliicoccaceae* but to bacteria within the order *Clostridiales*. In wild ptarmigans, the major genus within the order *Clostridiales* was *Acetobacterium*. A species of this genus, *Acetobacterium woodii*, has been shown to interact with methanogens in syntrophic relationships for the transfer of H2 and acetate, but to our knowledge no indications for syntrophic methylamine metabolism have been shown [66].

Production of methanol for methanol-using methanogens like *Methanomassiliicoccaceae* may result from the microbial degradation of pectin, a common component of the middle lamella of higher plants (e.g., in fruit peels) [67, 68]. Berries of *Empetrum nigrum* constitute one of the major food items during fall/winter for rock ptarmigans inhabiting northern Scandinavia [6]. *Salix polaris* and *Saxifraga* spp. are the main plant species consumed by Svalbard ptarmigans during these same seasons [7]. Both are dicotyledonous plants, which hypothetically account for the presence of genes for hydrolytic enzymes involved in pectin degradation in another arctic herbivore, the Svalbard reindeer (*Rangifer tarandus plathyrhyncus*) [31]. The presence of genes encoding for GH families and enzymes (e.g. pectinesterase) involved in pectin degradation supports the presence of pectin-degrading microbes. Taxonomic annotation of sequences for pectinesterase genes pointed to species within *Bacteroidetes* (mainly families *Bacteroidaceae* and *Prevotellaceae*) and *Firmicutes* (order *Clostridiales*) as the major pectin degraders in wild ptarmigans. Pectinolytic activity has been previously observed in several members of these bacterial groups [69, 70]. Genes related to methanol-based methanogenesis were identified in wild ptarmigans, almost exclusively assigned to *Methanomassiliicocacceae*, agreeing with the high abundances of *Methanomassiliicocacceae* observed (Table 1). We hypothesize that pectin metabolism in the cecum of ptarmigans leads to the formation of methanol that is utilized by *Methanomassiliicocacceae*-related methanogens. Methanogenesis from methanol originating from pectin degradation have been postulated from the study of frugivorous hindgut fermenters like the orangutan (*Pongo pygameus*), but with *Methanosphaera stadtmanae* being the major methanogens [71].

## Conclusions

It is concluded that wild ptarmigans in these two different geographical regions (Svalbard and Norway) share highly similar microbial communities. The presence of microbial taxa with the potential to decompose PSMs reflects an ability of ptarmigans to bypass the toxicity exerted by PSMs. The broad range of GHs found in wild and captive ptarmigans suggests the existence of a versatile cecum microbiota that allows the consumption of plants with different fiber contents. Methanol-based methanogenesis seems to constitute one of the major methanogenesis pathways in wild ptarmigans based on the dominance of *Methanomassiliicoccaceae* methanogens and the presence of genes for this pathway. The higher abundance of methanogens in wild ptarmigans may be linked to the ingestion of a natural diet consisting of unprocessed polysaccharides that result in longer fermentation necessary to allow growth of methanogenic Archaea.

## Supporting information

S1 Fig Schematic representation of the main pathways of methanogenesis and related enzymes based on KEGG-mapped genes found in the ceca of wild ptarmigans. Full name and KEGG entries (whenever possible) for the enzymes involved in each metabolic reaction (1–15) are given in the legend box. Red arrows indicate those substrates used for methane production alternative to the dominant H2-based methanogenesis. Red color numbers are used to highlight those enzymes characterized at a higher proportion in the cecal microbiome of wild ptarmigans used in this study compared to captive ptarmigans. Abbreviations: Fd_red_: reduced ferredoxin; Fd_ox_: oxidized ferredoxin; F_420_H_2_ reduced coenzyme F_420_; F_420_: oxidized coenzyme F_420_; MFR: methanofuran; H_4_MPT: tetrahydromethanopterin; CoM-SH: coenzyme M; CoB-SH: coenzyme B; CoM-S-S-CoB: heterodisulfide of CoM and CoB; SH-CoA: coenzyme A; CHO-MFR: formyl-MFR; CHO-H_2_MPT: formyl-H_4_MPT; CH-H_4_MPT: methenyl-H_4_MPT; CH_2_-H_4_MPT: methylene-H_4_MPT; CH_3_-H_4_MPT: methyl-H_4_MPT; CH_3_-SCoM: methyl-CoM; CH_3_-CO-S-CoA: methyl-AcylCoA; CO-S-CoA: acyl-CoA; CoA-SH: coenzyme A. * Electrons for the reduction of methyl groups are obtained by methyl-group oxidation to CO_2_ reversible to methanogenesis. Modified from [71].
(PDF)

S2 Fig Relative abundance of genes encoding hydrolytic enzymes in the cecum of wild and captive ptarmigans. Values are given as percentages of the total sequences assigned to protein family domains involved in polysaccharide degradation. Bar charts were scaled according to the relative abundance for each functional category. Identification of each domain was performed by BLAST against the Pfam-hmm database (see Methods for more details). a) Total results for the major functional categories; b) Celullases; c) Enzymes involved in starch degradation; d) Endohemicellulases; e) Debranching enzymes; f) Olygosasaccharides hydrolases (GHs accounting for <1% of total hits each were combined in a single group named “COMBINED GROUP GH <1%”for a better visualization).
(PDF)

S1 Table qRT-PCR conditions for amplification of methanogens and bacteria in cecum samples from wild and captive ptarmigans. (DOCX)

S2 Table Number of sequences per sample used for 16S rRNA gene amplicon sequencing analysis. (DOCX)

S3 Table Information on the shotgun metagenomics results on cecal samples from wild and captive ptarmigans. (DOCX)

S4 Table Total pfam families associated to the degradation of polysaccharides in cecal samples from wild ptarmigans. Results are represented as the percentage of sequences associated to a specific GH family in relation to the total pfam hits.
(DOCX)

S5 Table Bacterial and methanogens counts from different host animals. Numbers are presented with their corresponding units.
(DOCX)

S6 Table Total diversity of Glycoside Hydrolase (GH) families in several animal hosts. (DOCX)
