# Supplementary material for: Characterization of the cecum microbiome from wild and captive rock ptarmigans indigenous to Arctic Norway
Source: PLoS One. 2019 Mar 11;14(3):e0213503. doi: 10.1371/journal.pone.0213503 (PMC6411164; doi:10.1371/journal.pone.0213503)
Supplement: S1 Fig — Full name and KEGG entries (whenever possible) for the enzymes involved in each metabolic reaction (1–15) are given in the legend box. Red arrows indicate those substrates used for methane production alternative to the dominant H2-based methanogenesis. Red color numbers are used to highlight those enzymes characterized at a higher proportion in the cecal microbiome of wild ptarmigans used in this study compared to captive ptarmigans. Abbreviations: Fdred: reduced ferredoxin; Fdox: oxidized ferredoxin; F420H2 reduced coenzyme F420; F420: oxidized coenzyme F420; MFR: methanofuran; H4MPT: tetrahydromethanopterin; CoM-SH: coenzyme M; CoB-SH: coenzyme B; CoM-S-S-CoB: heterodisulfide of CoM and CoB; SH-CoA: coenzyme A; CHO-MFR: formyl-MFR; CHO-H2MPT: formyl-H4MPT; CH-H4MPT: methenyl-H4MPT; CH2-H4MPT: methylene-H4MPT; CH3-H4MPT: methyl-H4MPT; CH3-SCoM: methyl-CoM; CH3-CO-S-CoA: methyl-AcylCoA; CO-S-CoA: acyl-CoA; CoA-SH: coenzyme A. * Electrons for the reduction of methyl groups are obtained by methyl-group oxidation to CO2 reversible to methanogenesis. Modified from [71]. (PDF) [file pone.0213503.s001.pdf]

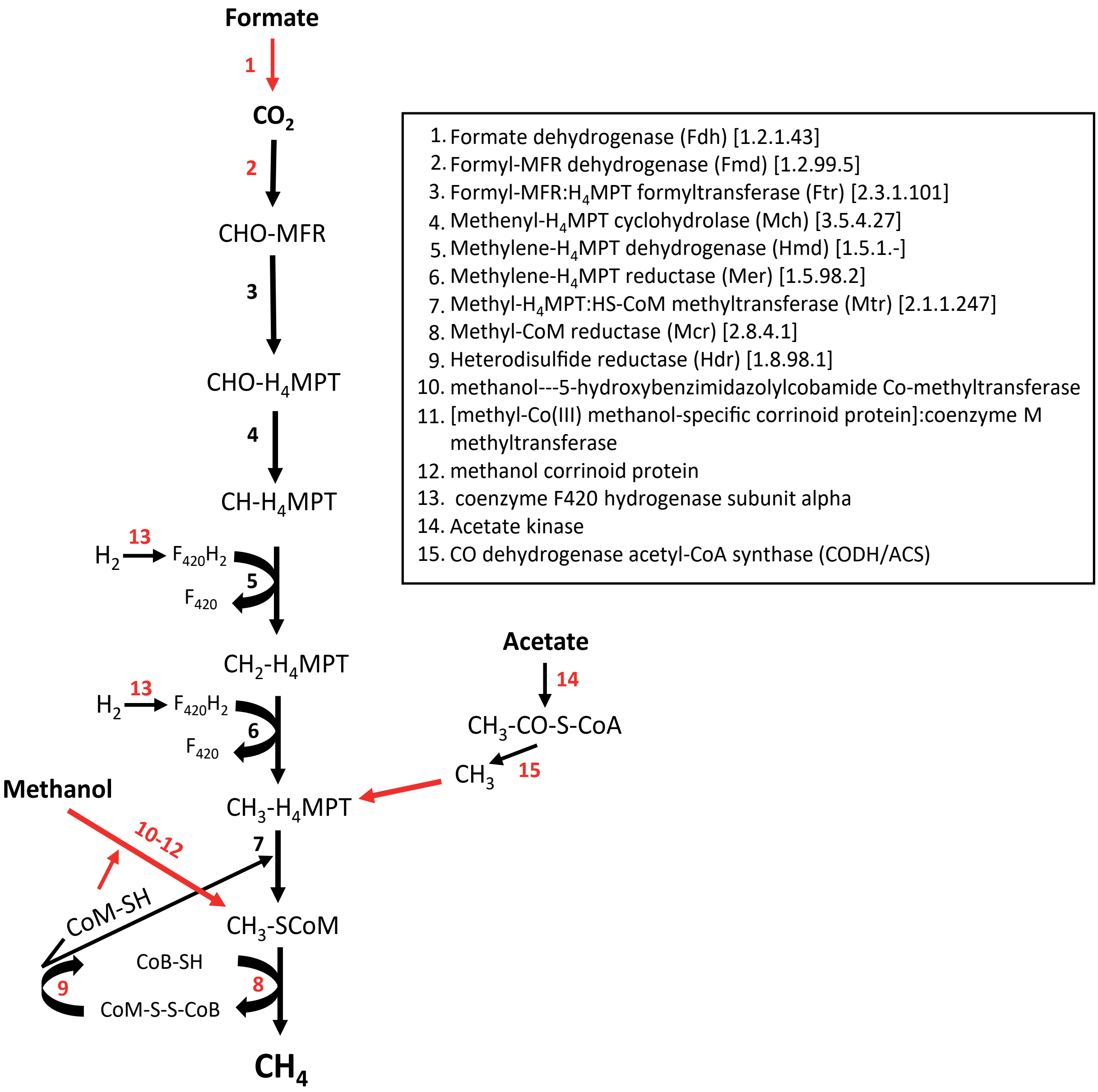
Formate
1
CO2
2
CHO-MFR
3
CHO-H4MPT
4
CH-H4MPT
H2
13
F420H2
5
F420
CH2-H4MPT
H2
13
F420H2
6
F420
Acetate
14
CH3-CO-S-CoA
CH3
15
Methanol
CH3-H4MPT
10-12
7
CoM-SH
CH3-SCoM
CoB-SH
9
8
CoM-S-S-CoB
CH4
1. Formate dehydrogenase (Fdh) [1.2.1.43]
2. Formyl-MFR dehydrogenase (Fmd) [1.2.99.5]
3. Formyl-MFR:H4MPT formyltransferase (Ftr) [2.3.1.101]
4. Methenyl-H4MPT cyclohydrolase (Mch) [3.5.4.27]
5. Methylene-H4MPT dehydrogenase (Hmd) [1.5.1.-]
6. Methylene-H4MPT reductase (Mer) [1.5.98.2]
7. Methyl-H4MPT:HS-CoM methyltransferase (Mtr) [2.1.1.247]
8. Methyl-CoM reductase (Mcr) [2.8.4.1]
9. Heterodisulfide reductase (Hdr) [1.8.98.1]
10. methanol---5-hydroxybenzimidazolylcobamide Co-methyltransferase
11. [methyl-Co(III) methanol-specific corrinoid protein]:coenzyme M methyltransferase
12. methanol corrinoid protein
13. coenzyme F420 hydrogenase subunit alpha
14. Acetate kinase
15. CO dehydrogenase acetyl-CoA synthase (CODH/ACS)
