# Supplementary figures and images for: Characterization of the cecum microbiome from wild and captive rock ptarmigans indigenous to Arctic Norway
Source: PLoS One. 2019 Mar 11;14(3):e0213503. doi: 10.1371/journal.pone.0213503 (PMC6411164; doi:10.1371/journal.pone.0213503)

A.

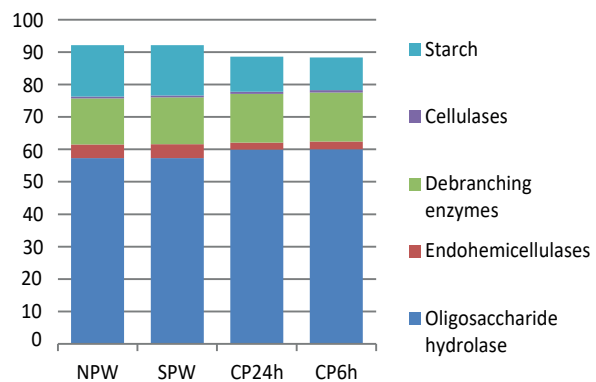

B.

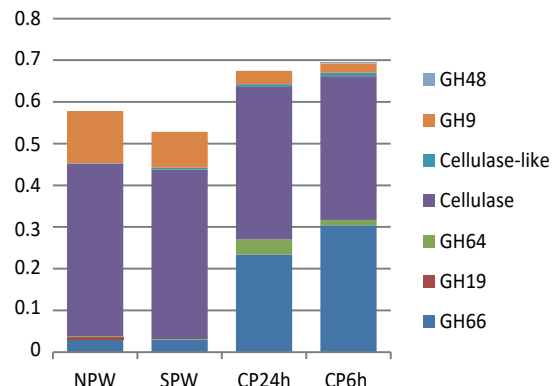

C.

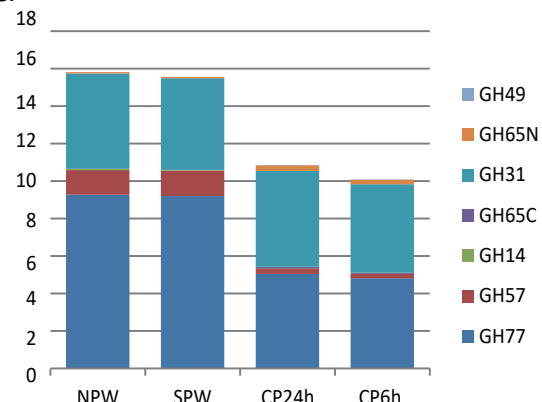

D.

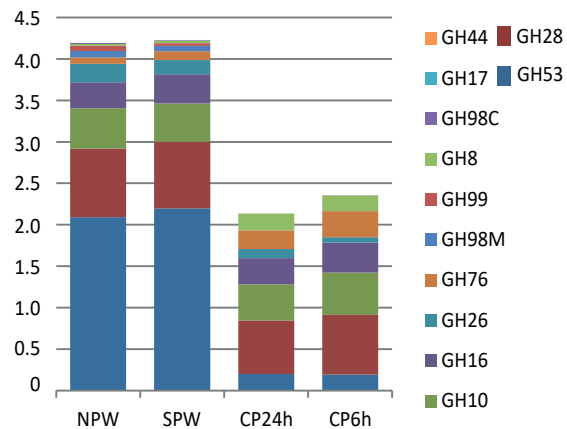

E.

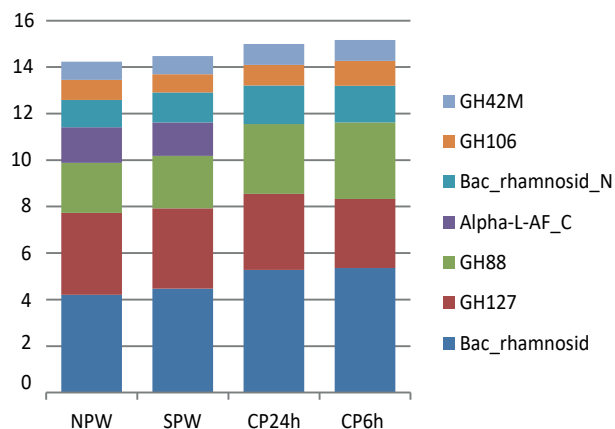

F.

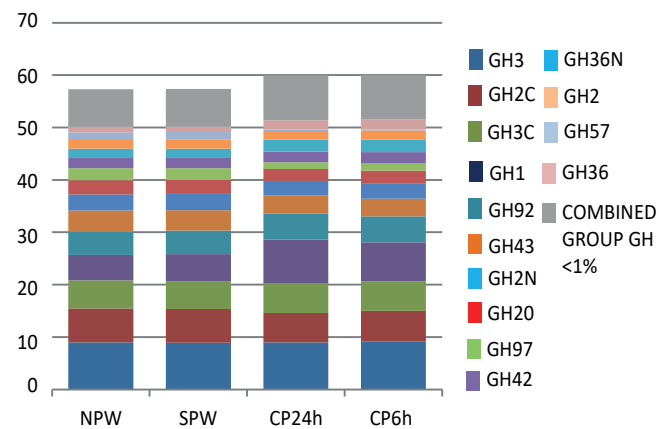

Supplement: S2 Fig — Values are given as percentages of the total sequences assigned to protein family domains involved in polysaccharide degradation. Bar charts were scaled according to the relative abundance for each functional category. Identification of each domain was performed by BLAST against the Pfam-hmm database (see Methods for more details). a) Total results for the major functional categories; b) Celullases; c) Enzymes involved in starch degradation; d) Endohemicellulases; e) Debranching enzymes; f) Olygosasaccharides hydrolases (GHs accounting for <1% of total hits each were combined in a single group named “COMBINED GROUP GH <1%”for a better visualization). (PDF) [file pone.0213503.s002.pdf]
