# Supplementary material for: Characterization of the cecum microbiome from wild and captive rock ptarmigans indigenous to Arctic Norway
Source: PLoS One. 2019 Mar 11;14(3):e0213503. doi: 10.1371/journal.pone.0213503 (PMC6411164; doi:10.1371/journal.pone.0213503)
Supplement: S1 Table — (DOCX) [file pone.0213503.s003.docx]

| **Microbial group** | **Initial denaturation** |  | **Denaturation** |  | **Annealing** |  | **Elongation** |  | **Cycles** | **Melting curve** |
| --- | --- | --- | --- | --- | --- | --- | --- | --- | --- | --- |
|  | **Temp. (°C)** | **Time** | **Temp. (°C)** | **Time** | **Temp. (°C)** | **Time** | **Temp. (°C)** | **Time** |  | **Temp. (°C)** |
| Bacteria | 95 | 15 sec. | 95 | 30 sec. | 60 | 30 sec. | 72 | 60 sec. | x40 | 60 to 95 (0.5°C/sec.) |
| Methanogens | 95 | 15 sec. | 95 | 30 sec. | 60 | 30 sec. | 72 | 30 sec. | x40 | 60 to 95 (0.5°C/sec.) |
