# Supplementary material for: Characterization of the cecum microbiome from wild and captive rock ptarmigans indigenous to Arctic Norway
Source: PLoS One. 2019 Mar 11;14(3):e0213503. doi: 10.1371/journal.pone.0213503 (PMC6411164; doi:10.1371/journal.pone.0213503)
Supplement: S2 Table — (DOCX) [file pone.0213503.s004.docx]

|  | **Bacteria** | **Archaea** |
| --- | --- | --- |
| **Samples** | **Nº of sequences** | **Nº of sequences** |
| SPW1 | 19774 | 24709 |
| SPW2 | 20528 | 20388 |
| SPW3 | 21093 | 27570 |
| SPW4 | 20197 | 10019 |
| NPW1 | 18456 | 2051 |
| NPW2 | 18558 | 2917 |
| NPW3 | 19011 | 1615 |
| NPW4 | 13245 | NA |
| **TOTAL** | **150862** | **89269** |
