# Supplementary material for: Characterization of the cecum microbiome from wild and captive rock ptarmigans indigenous to Arctic Norway
Source: PLoS One. 2019 Mar 11;14(3):e0213503. doi: 10.1371/journal.pone.0213503 (PMC6411164; doi:10.1371/journal.pone.0213503)
Supplement: S3 Table — (DOCX) [file pone.0213503.s005.docx]

| **Sample ID** | **Raw simple** | **After prinseq** | **Average length (bp)** | **Subset MEGAN** | **TOTAL hits** | **Taxonomy** | **KEGG** | **KEGG hits (%)** | **Pfam subsample** | **Hits Pfam** | **SSU rRNA** | **Assigned SSU rRNA** |
| --- | --- | --- | --- | --- | --- | --- | --- | --- | --- | --- | --- | --- |
| NPW1 | 48926599 | 26224335 | 267 | 5000000 | 3198538 | 3198393 | 1542694 | 48.2 | 5000000 | 1484201 | 26834 | 18878 |
| NPW2 | 42221650 | 22154168 | 268 | 5000000 | 3305314 | 3304755 | 1524948 | 46.1 | 5000000 | 1495362 | 28307 | 20797 |
| NPW3 | 36506282 | 18307405 | 270 | 5000000 | 2643880 | 2643408 | 1256817 | 47.5 | 5000000 | 1226039 | 33813 | 18531 |
| NPW4 | 43585955 | 22793216 | 267 | 5000000 | 3291822 | 3281343 | 1593924 | 48.4 | 5000000 | 1524782 | 27068 | 20520 |
| SPW1 | 41344835 | 20888176 | 269 | 5000000 | 3255531 | 3255049 | 1548320 | 47.5 | 5000000 | 1477622 | 25810 | 18075 |
| SPW2 | 43079396 | 21534125 | 269 | 5000000 | 3187758 | 3187194 | 1476291 | 46.3 | 5000000 | 1433812 | 28741 | 19229 |
| SPW3 | 41477964 | 20749128 | 269 | 5000000 | 2625781 | 2625358 | 1251518 | 47.7 | 5000000 | 1220810 | 32124 | 16991 |
| SPW4 | 46325111 | 23871798 | 267 | 5000000 | 3251942 | 3251386 | 1546102 | 47.5 | 5000000 | 1494005 | 25253 | 18114 |
| CP24h1 | 43991666 | 22366463 | 265 | 5000000 | 3923047 | 3925070 | 1512274 | 38.5 | 5000000 | 1693398 | 29096 | 23992 |
| CP24h2 | 54056433 | 23949951 | 273 | 5000000 | 3958839 | 3957792 | 1489513 | 37.6 | 5000000 | 1579394 | 33981 | 27362 |
| CP24h3 | 42858388 | 22056984 | 263 | 5000000 | 3819857 | 3818562 | 1449490 | 37.9 | 5000000 | 1632666 | 32087 | 26255 |
| CP24h4 | 48933015 | 24841714 | 265 | 5000000 | 3895527 | 3894851 | 1485459 | 38.1 | 5000000 | 1644431 | 29262 | 23610 |
| CP6h1 | 14200170 | 6816861 | 276 | 5000000 | 3477419 | 3474213 | 1498655 | 43.1 | 5000000 | 8196595 | 40512 | 33791 |
| CP6h2 | 43728552 | 22209260 | 266 | 5000000 | 3918861 | 3917861 | 1461281 | 37.3 | 5000000 | 1646706 | 30432 | 24699 |
| CP6h3 | 44169555 | 21730241 | 266 | 5000000 | 3864718 | 3863967 | 1510037 | 39.1 | 5000000 | 1681020 | 31199 | 24525 |
| CP6h4 | 16464264 | 8263885 | 264 | 5000000 | 3720305 | 3716261 | 1422564 | 38.2 | 5000000 | 1629102 | 30810 | 24668 |
