# Supplementary material for: Characterization of the cecum microbiome from wild and captive rock ptarmigans indigenous to Arctic Norway
Source: PLoS One. 2019 Mar 11;14(3):e0213503. doi: 10.1371/journal.pone.0213503 (PMC6411164; doi:10.1371/journal.pone.0213503)
Supplement: S4 Table — Results are represented as the percentage of sequences associated to a specific GH family in relation to the total pfam hits. (DOCX) [file pone.0213503.s006.docx]

| **pfam** | **function** | **NPW** | **SPW** | **Function** | **Functional group** |
| --- | --- | --- | --- | --- | --- |
| PF02446.14 | GH77 | 9.275 | 9.197 | 4-α-glucanotransferase/amylomaltase | Starch degradation |
| PF00933.18 | GH3 | 8.958 | 8.923 | β-glucosidase | Oligosaccharide hydrolase |
| PF02836.14 | GH2C | 6.510 | 6.451 | β-galactosidase | Oligosaccharide hydrolase |
| PF01915.19 | GH3C | 5.349 | 5.290 | β-glucosidase | Oligosaccharide hydrolase |
| PF00232.15 | GH1 | 4.860 | 5.146 | β-glucosidase | Oligosaccharide hydrolase |
| PF01055.23 | GH31 | 5.089 | 4.874 | α-glucosidase | Starch degradation |
| PF07971.9 | GH92 | 4.471 | 4.496 | α-mannosidase | Oligosaccharide hydrolase |
| PF05592.8 | Bac_rhamnosid | 4.203 | 4.473 | α-L-rhamnosidase | Debranching enzyme |
| PF04616.11 | GH43 | 3.992 | 3.918 | arabino/xylosidase | Oligosaccharide hydrolase |
| PF07944.9 | GH127 | 3.523 | 3.450 | β-L-arabinofuranosidase | Debranching enzyme |
| PF02837.15 | G2N | 3.089 | 3.093 | β-galactosidase | Oligosaccharide hydrolase |
| PF00728.19 | GH20 | 2.729 | 2.648 | β-hexasominidase | Oligosaccharide hydrolase |
| PF10566.6 | GH97 | 2.292 | 2.282 | α-glucosidase | Oligosaccharide hydrolase |
| PF07470.10 | GH88 | 2.157 | 2.257 | d-4.5-unsaturated β-glucuronyl hydrolase | Debranching enzyme |
| PF07745.10 | GH53 | 2.091 | 2.198 | endo-1.4-β-galactanases (endohemicellulases) | Endohemicellulase |
| PF02449.12 | GH42 | 1.906 | 1.930 | β-galactosidase | Oligosaccharide hydrolase |
| PF16875.2 | GH36N | 1.807 | 1.815 | α-galactosidase | Oligosaccharide hydrolase |
| PF00703.18 | GH2 | 1.819 | 1.773 | β-galactosidase | Oligosaccharide hydrolase |
| PF06964.9 | Alpha-L-AF_C | 1.535 | 1.441 | α-L-arabinofuranosidase | Debranching enzyme |
| PF03065.12 | GH57 | 1.315 | 1.350 | α-galactosidase/α-amylase | Oligosaccharide hydrolase |
| PF02922.15 | CBM_48 | 1.277 | 1.331 | Binding-module potentially associated to starch-debranching enzymes | Starch degradation |
| PF08531.7 | Bac_rhamnosid_N | 1.168 | 1.291 | α-L-rhamnosidase | Debranching enzyme |
| PF03629.15 | SASA | 1.031 | 1.186 | Carbohydrate esterase | Debranching enzyme |
| PF17167.1 | GH36 | 1.074 | 1.052 | α-galactosidase | Oligosaccharide hydrolase |
| PF15979.2 | GH115 | 0.939 | 0.883 | α-glucuronidase activity (hydrolysis of xylan) | Oligosaccharide hydrolase |
| PF01229.14 | GH39 | 0.879 | 0.935 | β-xylosidase | Oligosaccharide hydrolase |
| PF01301.16 | GH35 | 0.898 | 0.905 | β-galactosidase | Oligosaccharide hydrolase |
| PF17132.1 | GH106 | 0.869 | 0.786 | α-L-rhamnosidase | Debranching enzyme |
| PF00295.14 | GH28 | 0.825 | 0.805 | polygalacturonase | Endohemicellulase |
| PF08532.7 | GH42M | 0.776 | 0.781 | β-galactosidase/L-arabinofuranosidase | Debranching enzyme |
| PF04041.10 | GH130 | 0.610 | 0.625 | phosphorilase of β-mannosinic | - |
| PF02056.13 | GH4 | 0.584 | 0.594 | α- β- glucosidase/α-galacturonase | Oligosaccharide hydrolase |
| PF16874.2 | GH36C | 0.567 | 0.600 | α-galactosidase | Oligosaccharide hydrolase |
| PF02838.12 | GH20b | 0.525 | 0.536 | β-N-acetylglucosaminidases | Oligosaccharide hydrolase |
| PF01074.19 | GH38 | 0.526 | 0.467 | α-mannosidase | Oligosaccharide hydrolase |
| PF11975.5 | GH4C | 0.514 | 0.453 | α- β- glucosidase | Oligosaccharide hydrolase |
| PF00331.17 | GH10 | 0.487 | 0.460 | endo-1.4-β-xylanases | Endohemicellulase |
| PF07488.9 | GH67M | 0.418 | 0.463 | α-glucuronidase activity (hydrolysis of xylan) | Oligosaccharide hydrolase |
| PF00150.15 | Cellulase | 0.414 | 0.406 | Cellulase | Cellulases |
| PF07477.9 | GH67C | 0.358 | 0.368 | α-glucuronidase activity | Oligosaccharide hydrolase |
| PF02055.13 | GH30 | 0.343 | 0.373 | glucuronoxylan | Oligosaccharide hydrolase |
| PF00722.18 | GH16 | 0.315 | 0.352 | Transglycosylases | Endohemicellulase |
| PF07748.10 | GH38C | 0.292 | 0.287 | α-mannosidase | Oligosaccharide hydrolase |
| PF16011.2 | CBM9_2 | 0.291 | 0.258 | endoxylanase-like protein | Endohemicellulase |
| PF01532.17 | GH47 | 0.197 | 0.231 | α-mannosidase | Oligosaccharide hydrolase |
| PF03632.12 | GH65m | 0.196 | 0.207 | phosphorilases (maltose. trehalose) | - |
| PF05838.9 | GH108 | 0.205 | 0.196 | N-acetylmuramidase | lysozime activity |
| PF02156.12 | GH26 | 0.222 | 0.171 | endo-β-1.4-mannanases | Endohemicellulase |
| PF00404.15 | Dockerin_1 | 0.153 | 0.179 | Cellulosomal scaffolding proteins | Cellulosome |
| PF17189.1 | GH30C | 0.155 | 0.165 | glucuronoxylan | hemicellulose degradation |
| PF16760.2 | CBM53 | 0.122 | 0.124 | Carbohydrate -binding module | - |
| PF08244.9 | GH32C | 0.124 | 0.100 | invertase | - |
| PF00759.16 | GH9 | 0.125 | 0.086 | cellulase | Cellulases |
| PF03663.11 | GH76 | 0.079 | 0.107 | α-1.6-mannanases | Endohemicellulase |
| PF03422.12 | CBM_6 | 0.092 | 0.058 | Carbohydrate -binding module | - |
| PF00963.15 | Cohesin | 0.078 | 0.067 | Cellulosomal scaffolding proteins | Cellulosome |
| PF08306.8 | GH98M | 0.076 | 0.062 | endo-β-galactosidase | Endohemicellulase |
| PF02018.14 | CBM4_9 | 0.067 | 0.071 | Carbohydrate -binding module | - |
| PF03636.12 | GH65N | 0.064 | 0.066 | trehalose/maltose phosphorilase | Starch degradation |
| PF11790.5 | GHcc | 0.062 | 0.057 | putative glycoside hydrolase | - |
| PF08533.7 | GH42C | 0.058 | 0.056 | β-galactosidase | Oligosaccharide hydrolase |
| PF06452.8 | CBM9_1 | 0.055 | 0.057 | Carbohydrate -binding module | - |
| PF01373.14 | GH14 | 0.057 | 0.043 | β-amylase | Starch degradation |
| PF16317.2 | GH99 | 0.060 | 0.035 | endo-α-mannosidase | Endohemicellulase |
| PF13199.3 | GH66 | 0.029 | 0.029 | Dextranase | - |
| PF01270.14 | GH8 | 0.024 | 0.033 | endoglucanase | Endohemicellulase |
| PF11308.5 | GH129 | 0.023 | 0.019 | endo-α-N-acetylgalactosaminidase | - |
| PF16738.2 | CBM26 | 0.019 | 0.022 | Starch-binding protein | Starch degradation |
| PF03200.13 | GH63 | 0.025 | 0.015 | Mannosyl glucosidase | Oligosaccharide hydrolase |
| PF03442.11 | CBM_X2 | 0.022 | 0.017 | Carbohydrate-binding protein | - |
| PF02324.13 | GH70 | 0.013 | 0.019 | Glucosyltransferases | - |
| PF03633.12 | GH65C | 0.010 | 0.016 | trehalose/maltose phosphorilase | Starch degradation |
| PF01630.15 | GH56 | 0.008 | 0.013 | Hyaluronidase | - |
| PF00723.18 | GH15 | 0.012 | 0.008 | α-glucosidase | Oligosaccharide hydrolase |
| PF08307.8 | GH98C | 0.014 | 0.005 | endo-β-galactosidase | Endohemicellulase |
| PF02057.12 | GH59 | 0.008 | 0.009 | galactocerebrosidase | - |
| PF03644.10 | GH85 | 0.006 | 0.008 | endo-β-N-acetylglucosaminidase | - |
| PF00182.16 | GH19 | 0.006 | 0.000 | chitinases | - |
| PF12876.4 | Cellulase-like | 0.001 | 0.004 | Cellulase | Cellulases |
| PF16483.2 | GH64 | 0.002 | 0.002 | β-1.3-glucanase | Cellulases |
| PF03648.11 | GH67N | 0.004 | 0.000 | α-glucuronidase | Oligosaccharide hydrolase |
| PF16923.2 | GH63N | 0.001 | 0.000 | Mannosyl glucosidase | Oligosaccharide hydrolase |
| PF03512.10 | GH52 | 0.000 | 0.001 | β-xylosidase | Oligosaccharide hydrolase |
| PF12905.4 | GH101 | 0.001 | 0.000 | endo-β-N-acetylglucosaminidase | - |
