# Supplementary material for: Characterization of the cecum microbiome from wild and captive rock ptarmigans indigenous to Arctic Norway
Source: PLoS One. 2019 Mar 11;14(3):e0213503. doi: 10.1371/journal.pone.0213503 (PMC6411164; doi:10.1371/journal.pone.0213503)
Supplement: S5 Table — Numbers are presented with their corresponding units. (DOCX) [file pone.0213503.s007.docx]

| **Animal** | **Bacteria** | **Methanogens** | **Diet** | **Body part** | **Reference** |
| --- | --- | --- | --- | --- | --- |
| Svalbard ptarmigan (*Lagopus muta hyperboreus*) | 1.38×10^9^  (16S rRNA gene copies/gww) | 5.31×10^7^  (16S rRNA gene copies/gww) | Leaves (*Salix polaris & Saxifraga cespitosa*), berries (*Empetrum nigrum*) | Cecum | This study |
| Svalbard ptarmigan (*Lagopus muta hyperboreus*) (captive) | 1.40×10^9^  (16S rRNA gene copies/gww) | 4.73×10^7^  (16S rRNA gene copies/gww) | Leaves (*Salix polaris & Saxifraga cespitosa*), berries (*Empetrum nigrum*) | Cecum | This study |
| Norwegian rock ptarmigan (*Lagopus muta muta*) | 9.28×10^8^  (16S rRNA gene copies/gww) | 6.47×10^5^  (16S rRNA gene copies/gww) | Commerical pelleted feed | Cecum | This study |
| Brahman-crossbred steer (*Bos indicus*) | NA | 1.34×10^9^ (cells/mL) | Grass-based | Rumen | Denman et al. 2007 |
| Norwegian reindeer (*Rangifer tarandus tarandus*) | 5.17×10^11^  (cells/gww) | 3.17×10^9^  (cells/gww) | Summer pastures | Rumen | Sundset et al. 2009 |
| King penguin (*Aptenodytes patagonicus*) | 6.21×10^7^ CFU/gww | NA | Fish, squid | Feces | Dewar et al. 2013 |
| Gentoo penguin (*Pygoscelis papua*) | 6.05×10^7^ CFU/gww | NA | Fish, crustacean, squid | Feces | Dewar et al. 2013 |
| Macaroni penguin (*Eudyptula chrysolophus*) | 1.08×10^8^ CFU/gww | NA | Fish, crustacean, squid | Feces | Dewar et al. 2013 |
| Little penguin (*Eudytula minor*) | 1.46×10^6^ CFU/gww | NA | Fish, crustacean, squid | Feces | Dewar et al. 2013 |
| Broiler chicken | 1.08×10^11^ (16S rRNA gene copies/gww) | NA | Pelleted | Cecum | Mignon-Gastreau et al. 2015 |
| Swedish dairy cow | NA | 5.06×10^8^ (16S rRNA gene copies/mL) | Forage/concentrate (500/500) | Rumen | Danielsson et al. 2012 |
| Swedish dairy cow | NA | 2.88×10^7^ (16S rRNA gene copies/mL) | Forage/concentrate (900/100) | Rumen | Dannielson et al. 2012 |
| Leghorn chicken | NA | 4.51×10^7^ (16S rRNA gene copies/gww) | Commerical pelleted feed | Cecum | Saengkerdsub et al. 2007 |
| Hoatzin (*Opisthocomus hoazin*) | 7.93×10^12^ | 5.80×10^9^ (cells/gww) | Leaves, flowers, fruit | Crop | Wright et al. 2009 |
