# Supplementary material for: Characterization of the cecum microbiome from wild and captive rock ptarmigans indigenous to Arctic Norway
Source: PLoS One. 2019 Mar 11;14(3):e0213503. doi: 10.1371/journal.pone.0213503 (PMC6411164; doi:10.1371/journal.pone.0213503)
Supplement: S6 Table — (DOCX) [file pone.0213503.s008.docx]

| Animal | Total different GHs | Diet | Body part | Reference |
| --- | --- | --- | --- | --- |
| Wild rock ptarmigan (Norwegian & Svalbard) | 89 | Leaves (Salix polaris & Saxifraga cespitosa), berries (Empetrum nigrum) | Cecum | This study |
| Svalbard rick ptarmigan (captive) | 91 | Commercial pelleted feed | Cecum | This study |
| Tammar Wallaby (Macropus eugenii) ^a^ | 53 | Grass + pelleted | Foregut | Pope et al. 2010 |
| Svalbard reindeer (Rangifer tarandus platyrhynchus) ^a^ | 30 | Winter pasture | Rumen | Pope et al. 2012 |
| Asian elephant (Elephas maximus) ^a^ | 82 | Grass, leaves, fruit | Feces | Ilmberger et al. 2014 |
| Simmental Angus cross breed ^a^ | 33 | Grass + legume | Rumen (liquid) | Brulc at al. 2009 |

^a^ GHs numbers as presented in Table 2 in Ilmberger et al. 2014.
